# Comparison of Cervical Spine Kinematics and Clinical Neck Symptoms Between Mobile Device and Desktop Computer Use

**DOI:** 10.3390/s25051438

**Published:** 2025-02-26

**Authors:** Myung Woo Park, Min Yong Seong, Young Seop Song, Kibum Youn, Kyung Yong Yang, Jehee Lee, Sun Gun Chung, Keewon Kim

**Affiliations:** 1 Department of Rehabilitation Medicine, Seoul National University College of Medicine, Seoul Metropolitan Government-Seoul National University Boramae Medical Center, Seoul 07061, Republic of Korea; bmw.snu@gmail.com; 2 Seosong Hospital, Incheon 21043, Republic of Korea; 3 Seoul Gangnam Rehabilitation Clinic KR, Seoul 06275, Republic of Korea; 4 DIDIM Inc., Seongnam 13605, Republic of Korea; 5 Samsung Life Insurance, Seoul 06620, Republic of Korea; 6 School of Computer Science and Engineering, Seoul National University, Seoul 08826, Republic of Korea; 7 Department of Rehabilitation Medicine, Seoul National University College of Medicine, Seoul National University Hospital, Seoul 03080, Republic of Korea

**Keywords:** mobile device, desktop computer, neck pain, kinematics

## Abstract

The widespread use of mobile devices and desktop computers has been associated with mechanical neck symptoms, yet few studies have compared cervical kinematics and clinical symptoms between them. In this study, 15 participants (27.7 ± 4.4 years, 12 male) performed four randomly ordered 20 min tasks: two mobile (smartphone and tablet) and two desktop computer (keyboard and mouse) tasks. Kinematic variables, including neck flexion, lateral bending, axial rotation, anterior translation, and total distance moved, were measured using an optical motion capture system, while clinical symptoms, including discomfort, pain, tension, and fatigue, were assessed using a visual analog scale. Paired t-tests and linear mixed models were used for analysis. Results showed that mobile device users exhibited greater neck flexion (38.9° [32.1–45.6°] vs. −0.2° [−4.3–3.9°], *p* < 0.001) and anterior translation (21.0 cm [12.0–30.1] vs. 1.6 cm [−4.4–7.7], *p* < 0.001) compared to desktop users. All clinical symptoms were significantly higher during mobile device use (*p* < 0.05), with neck flexion and anterior translation strongly correlating with symptom severity. In conclusion, mobile device use leads to more severe neck symptoms compared to desktop computer use, which is associated with increased flexion and forward head posture. To reduce neck symptoms, avoiding sustained flexion and forward head positions during mobile device use is recommended.

## 1. Introduction

The use of desktop computers is well-known as a substantial risk factor for work-related musculoskeletal neck symptoms [1,2,3,4], which are associated with improper ergonomic practices and postures [5]. Workers tend to excessively increase forward head posture and neck flexion while working on desktop computers, with angles ranging from neutral to 50° depending on the workstation setup [6,7,8]. This posture can increase the load on cervical discs and activate neck muscles, further exacerbating the strain on the cervical spine [6,9,10]. In addition to desktop computers, mobile devices such as smartphones and tablets have also become widely adopted over the past years [11]. Many users adopt a forward head posture while using these devices, particularly while sitting, which can result in non-neutral neck positions [12]. This posture commonly results from holding mobile devices below eye level, causing users to tilt their heads forward to view the screen [13,14]. Factors such as screen size and task type can further influence neck posture [15,16]. Prolonged use of mobile devices has been associated with an increased prevalence of musculoskeletal symptoms, particularly neck pain [17,18,19,20,21]. Studies have shown that smartphone use often involves significant neck flexion, with angles ranging from 33° to 45°, leading to increased strain on the cervical spine [13,14]. Additionally, the repetitive use of smartphones for tasks such as texting and browsing exacerbates the strain on neck muscles and contributes to the development of neck pain [22].

Previous ergonomic studies have compared computer and mobile device use, with some finding that personal digital assistant (PDA) users were more likely to adopt a flexed neck posture compared to laptop users, with a notable proportion of users reporting discomfort [23]. Additionally, tablet use has been associated with greater neck flexion compared to viewing a monitor on a table [24,25]. However, many of these studies have focused on relatively short task durations, such as 1 to 15 min, or involved activities like watching movies rather than work-related tasks. Research that simultaneously evaluates cervical kinematics and clinical symptoms during prolonged use of mobile devices and computers in real-world, non-ergonomic settings is still limited.

Given the wide variety of mobile devices and the different modes of computer use—such as the predominant use of a mouse versus a keyboard—the impact on cervical kinematics may vary some extent. Moreover, neck pain can manifest in various ways depending on its underlying etiology [26], with clinical symptoms ranging from discomfort and stiffness to fatigue, rather than just pain alone [27]. Evaluating neck symptoms solely based on pain intensity may overlook the diverse manifestations. Therefore, it is essential to assess a broader spectrum of clinical symptoms, including qualitative aspects, related to both mobile device and computer use [27]. Studies that consider these factors and evaluate cervical kinematics and clinical symptoms across different device types and usage patterns are needed to better understand their relationship in real-world settings.

Given these considerations, the aim of this study was to compare cervical kinematics and clinical neck symptoms between mobile device and desktop computer use and to investigate their association across different tasks.

## 2. Materials and Methods

### 2.1. Participants

A total of fifteen healthy adults (mean age: 27.7 ± 4.4 years; mean height: 171.1 ± 5.7 cm; 12 men and 3 women) were recruited through poster advertisements at local universities. Inclusion criteria included age ≥18 years and at least one year of experience using both mobile devices and desktop computers. Participants were excluded if they had a history of neck disorders associated with pain or limitation in cervical spine range of motion, such as cervical trauma, herniated intervertebral disc, congenital or acquired cervical deformity, cervical tumors, infections, neuromuscular diseases, or a history of cervical spine surgery. Additionally, individuals with cervical range of motion limitations, as determined by an independent musculoskeletal rehabilitation physician, were excluded following a physical examination. This research was approved by the Institutional Review Board of Seoul National University Hospital (No. H-1210-007-431). Informed consent was obtained from all individual participants.

### 2.2. Experimental Protocols

**Figure 1:**
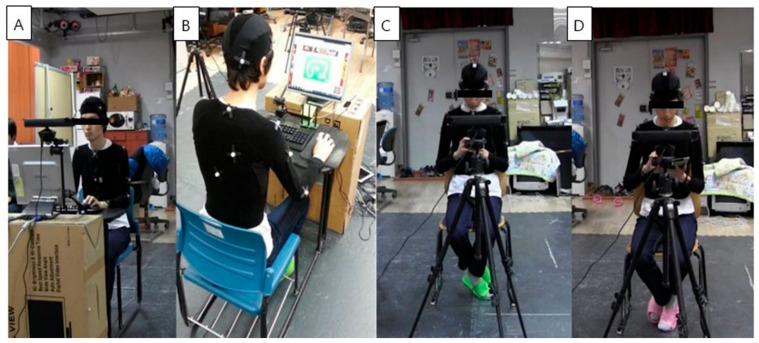
(**A**) Participant typing on a keyboard. (**B**) Participant using a mouse to play a game. (**C**) Participant playing a game on a smartphone. (**D**) Participant playing a game on a tablet.

Participants performed four randomly ordered tasks for 20 min each in a room equipped with optical motion capture cameras. The tasks were divided into two categories: mobile device usage and desktop computer usage. Mobile devices included smartphones (Apple iPhone 6, Apple, Inc., Cupertino, CA, USA) and tablets with touchscreen interfaces (Apple iPad 4, Apple, Inc., Cupertino, CA, USA), while desktop tasks were performed using a keyboard and mouse. To minimize potential carryover effects, the tasks were each performed on separate days and were consistently scheduled in the afternoon. To simulate real-world usage scenarios, participants were seated in chairs with a seat height of 44 cm, equipped with back support and without armrests. For the keyboard and mouse tasks, the height of the keyboard was adjusted to ensure that the participants’ elbows remained at a right-angle during task performance. The height and inclination of the monitor were also adjustable, allowing participants to set the top of the screen below horizontal eye level based on their comfort. Participants were instructed to assume a comfortable posture without specific guidelines regarding the positioning of the mobile device relative to their bodies or restrictions on gaze direction during the task. The keyboard task involved typing in a practice program, the mouse task involved playing the game Labyrinth2 (Illusions Labs, Malmö, Sweden), and the mobile device task involved playing the game Plants vs. Zombies (Electronic Arts, Redwood City, CA, USA) (Figure 1).

### 2.3. Kinematic Analysis

#### 2.3.1. Optical Motion Capture System

A VCam system (Vicon, Oxford, UK) was composed of 14 cameras with a 0.3-megapixel resolution that captured 15 optical markers at 30 fps. The markers were placed following the upper body modeling with the Plug-in Gait protocol: four head markers, five trunk markers, and six upper limb markers [28]. Head markers included two frontal and two back markers placed on the temples and posterior aspect of the head along the same transverse plane. Trunk markers were placed at the 7th cervical and 10th thoracic vertebrae, right scapula, jugular notch, and xiphoid process of the sternum. Upper limb markers were placed at the acromioclavicular joints, upper lateral arm, and lateral epicondyles.

#### 2.3.2. Data Analysis

The orientations and locations of the bodies and the heads of the participants were defined based on three-dimensional (3D) coordinates of body parts captured by the motion capture system. Neck posture was modeled as two rigid body segments (head and body) connected by a six-degrees-of-freedom joint. The motion between these segments was described using a rotation matrix and a translation matrix. The following equation represents the transformation of coordinates from the body frame to the head frame:

$$\left[\begin{array}{cc} R_{b}\left(t\right) & T_{b}\left(t\right)\\ 0 & 1 \end{array}\right]\left[\begin{array}{cc} R_{1} & T_{1}\\ 0 & 1 \end{array}\right]\left[\begin{array}{cc} R_{n}\left(t\right) & T_{n}\left(t\right)\\ 0 & 1 \end{array}\right]\left[\begin{array}{cc} R_{2} & T_{2}\\ 0 & 1 \end{array}\right]=\left[\begin{array}{cc} R_{h}\left(t\right) & T_{h}\left(t\right)\\ 0 & 1 \end{array}\right]$$

In this equation, *b* represents the body, *n* the neck joint, and *h* the head. The subscripts 1 and 2 indicate the transformations from the body frame to the neck frame and from the neck frame to the head frame, respectively. The rotation matrices $R_{1}$ and $R_{2}$ were derived from the initial neck orientation $R_{n}\left(0\right)$, which was aligned with the sagittal, coronal, and transverse planes. The translation matrices $T_{1}$ and $T_{2}$ were calculated from the participants’ range of motion data, with the minimization of $\left|\left|T_{n}\left(t\right)\right|\right|$ to ensure accurate motion capture. The resulting neck motion was converted into Euler angles, providing detailed clinical measurements for flexion/extension, left/right bending, and axial rotation. Anterior translational movement was derived from the translation matrix *T*.

#### 2.3.3. Kinematic Variables

**Figure 2:**
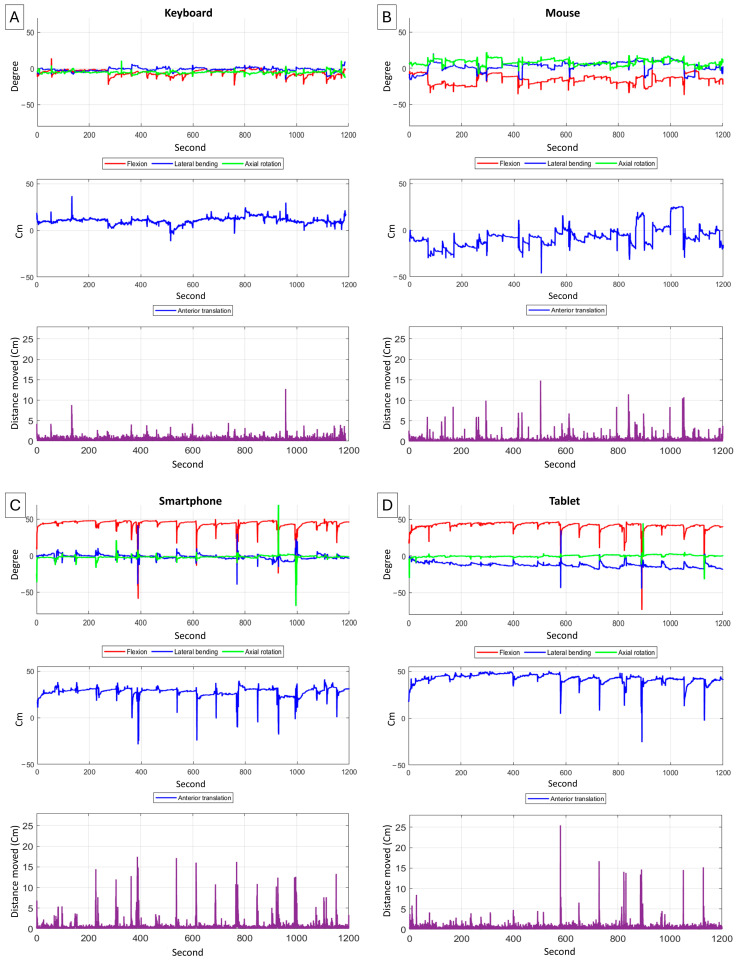
Kinematic data of one participant over 20 min for each task: (**A**) keyboard, (**B**) mouse, (**C**) smartphone, and (**D**) tablet. The first row shows kinematic variables: flexion (red), lateral bending (blue), and axial rotation (green). The second row represents anterior translation, while the third row illustrates the total distance moved.

We measured five kinematic variables: flexion, lateral bending, axial rotation, anterior translation, and distance moved. Flexion, lateral bending, and axial rotation were defined as the head’s relative rotation to the trunk in the sagittal, coronal, and transverse planes, respectively, from a neutral posture. Anterior translation was defined as the forward movement of the head relative to the trunk in the sagittal plane. Distance moved was the total length of the head’s trajectory during the task, calculated as the sum of absolute position changes relative to the trunk in 3D coordinates. Flexion was considered positive when the neck was flexed, anterior translation when the head moved forward, lateral bending when the neck tilted left, and axial rotation when the head turned right. For lateral bending and axial rotation, absolute values were used since the direction of motion did not affect mechanical stress on the neck. Angles were measured in degrees and distances in centimeters (Figure 2).

### 2.4. Clinical Variables

We assessed four clinical neck symptoms: discomfort, pain, tension, and fatigue. Discomfort was defined as any unpleasant sensation, including pain or tension. Pain was described as a sharp or aching sensation localized to the neck, while tension referred to a feeling of tightness or stiffness around the neck muscles. Fatigue was defined as a generalized feeling of tiredness following task completion. Pain, tension, and discomfort were measured before and after each task to capture changes during task performance, while fatigue was evaluated only after task completion to assess cumulative fatigue. Fatigue at baseline was considered 0. The severity of all symptoms was rated using a 100 mm visual analog scale (VAS), where 0 mm represented no symptom (normal), and 100 mm indicated the worst imaginable symptom.

### 2.5. Statistical Analysis

Paired *t*-tests were used to compare kinematic variables between mobile device (smartphone and tablet) and desktop computer (keyboard and mouse) usage, as well as between individual devices within each category. Data normality was evaluated using the Shapiro–Wilk test. For clinical variables, a linear mixed model was employed to compare mobile device and desktop computer usage, with pre-task values used as covariates for discomfort, pain, and tension. For fatigue, only post-task values were analyzed using the linear mixed model. Device type (mobile vs. desktop) was included as a fixed effect, and least squares means (LSMs) were calculated for each device type (mobile and desktop) based on this model. These LSM estimates represent adjusted averages that take into account the influence of the pre-task values and differences between individual devices (e.g., smartphone, tablet, keyboard, and mouse). The total value for each category (mobile or desktop) was derived from these LSM estimates to reflect an overall comparison between the two types of devices. Device type (mobile vs. desktop) was included as a fixed effect to account for potential differences between the two. The association between kinematic and clinical variables was also evaluated using a linear mixed model, with data from both device types combined into one model as well. All tests were two-sided, and *p*-values < 0.05 were considered statistically significant. All statistical analyses were performed using SAS version 9.4 (SAS Institute, Cary, NC, USA).

## 3. Results

### 3.1. Kinematic Variables According to Task Type

**Figure 3:**
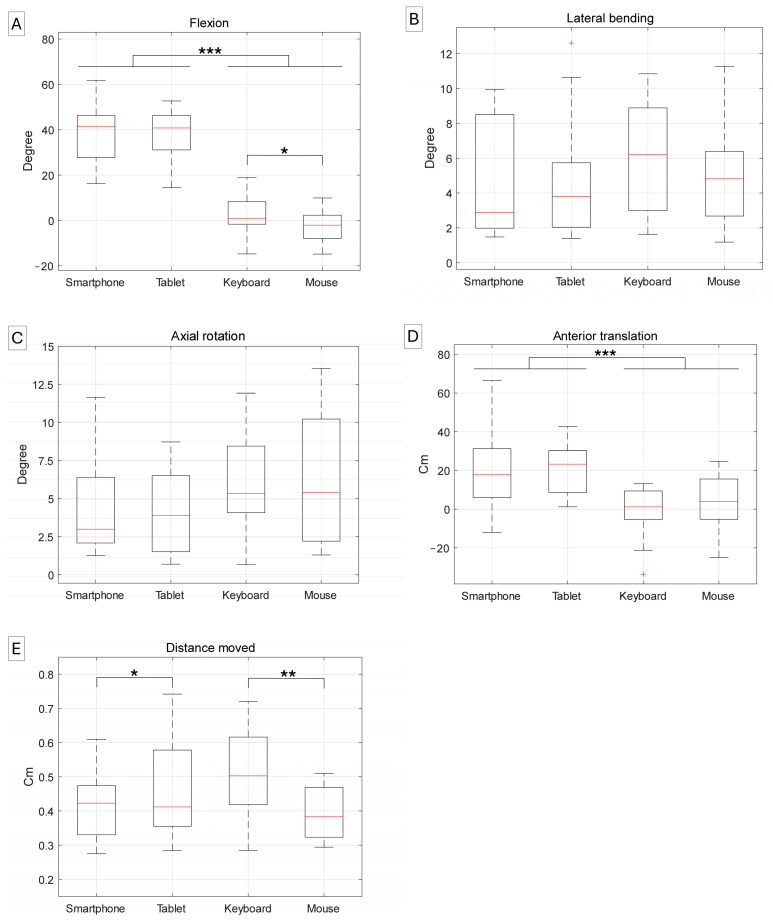
Kinematic variables for neck postures during tasks using mobile devices (smartphone and tablet) and a desktop computer (keyboard and mouse). (**A**) Flexion, (**B**) lateral bending, and (**C**) axial rotation were measured as the relative orientation of the head to the trunk in the sagittal, coronal, and transverse planes, respectively. (**D**) Anterior translation was calculated as the forward distance moved by the head relative to the trunk in the sagittal plane. (**E**) Distance moved represents the total head trajectory during each task. * *p* < 0.05, ** *p* < 0.01, and *** *p* < 0.001.

Mobile device usage was associated with greater flexion and anterior translation compared with desktop computer usage. Flexion during mobile device use was 38.9° (95% CI, 32.1° to 45.6°) compared with −0.2° (95% CI, −4.3° to 3.9°) during desktop computer use. Anterior translation was also greater with mobile devices, measuring 21.0 cm (95% CI, 12.0 to 30.1), compared with 1.6 cm (95% CI, −4.4 to 7.7) for desktop computers. There was no significant difference in flexion or anterior translation between smartphone and tablet use. Flexion differed significantly between keyboard and mouse use, though the difference was less than 5° [keyboard: 2.2° (95% CI, −2.5° to 6.9°); mouse: −2.5° (95% CI, −7.3° to 2.2°)]. No significant difference was observed in anterior translation between keyboard and mouse use. There were no differences in lateral bending, axial rotation, or distance moved between mobile device and desktop computer usage. For distance moved, significant differences were observed between smartphone and tablet usage [smartphone: 123 cm (95% CI, 105 to 144); tablet: 141 cm (95% CI, 120 to 162)], as well as between keyboard and mouse usage [keyboard: 153 cm (95% CI, 135 to 171); mouse: 117 cm (95% CI, 99 to 135)] (Figure 3).

### 3.2. Differences in Clinical Variables by Task Type

Mobile device usage was associated with higher scores in all clinical variables compared with desktop computer usage. No statistically significant differences were observed in clinical variables between smartphone and tablet usage. However, mouse usage was associated with significantly greater discomfort and fatigue compared with keyboard usage (Table 1).

### 3.3. Differences in Clinical Variables by Kinematics

The linear mixed model revealed that all post-task clinical variables significantly increased with flexion and anterior translation but not with lateral bending or axial rotation, irrespective of task type (Table 2).

## 4. Discussion

In the present study, the use of mobile devices was associated with significantly greater neck flexion and anterior translation compared to desktop computer tasks. The total distance moved was significantly greater with keyboard tasks compared to mouse tasks and with tablet use compared to smartphone use. Additionally, all clinical symptoms were significantly higher during mobile device use compared to desktop computer use and demonstrated a significant positive correlation with both neck flexion and anterior translation irrespective of tasks.

The findings of this study, showing increased neck flexion and anterior translation with mobile device use compared to desktop computer use, as well as heightened clinical symptoms, are consistent with, yet expand upon, previous studies that have explored the effects of different devices on cervical kinematics and clinical symptoms [17,18,19,20,21,24,25,29]. One study using surveys and laboratory experiments among laptop and PDA users found that a higher proportion of PDA users adopted a flexed neck posture compared to laptop users, with 21% of them reporting discomfort [23]. Another study, which compared muscle activity during smartphone texting and computer typing in young people with and without neck pain, confirmed that smartphone texting was associated with higher muscle activity in the cervical erector spinae muscles than computer typing. However, neither group showed a significant difference in discomfort scores in this study [30]. Similarly, another study observed significantly greater cervical flexion during smartphone texting compared with computer typing yet found no significant differences in discomfort scores between the groups [16]. The discrepancy between the results of previous studies and those of our study regarding clinical symptoms can be explained by two key factors. First, previous studies primarily focused on specific aspects of neck pain, such as location, severity, or duration, rather than incorporating a broader range of clinical symptoms. In our study, we aimed to capture a more comprehensive spectrum of symptoms by categorizing them into discomfort, pain, tension, and fatigue. This allowed for a more sensitive assessment of various dimensions of clinical neck symptoms, building on previous research that emphasized the importance of qualitative assessment in neck pain evaluation [27]. Second, participants in our study performed each task for 20 min, a longer duration compared to previous studies that evaluated durations of 1 to 15 min [23,24,25]. This extended time was chosen to better reflect real-world conditions and to capture the development of clinical symptoms more effectively. Additionally, all tasks were conducted on separate days to avoid any carryover effects or interference. Previous studies have shown a significant association between the time spent using mobile devices and the development of neck pain, which may have contributed to the differing outcomes in clinical symptoms observed in our study compared to earlier research [17,21].

The notable findings of this study included the fact that the total distance moved during the task was significantly different between mouse and keyboard tasks [keyboard = 153 cm (95% CI, 135–171); mouse = 117 cm (95% CI, 99–135)], with significantly greater discomfort and fatigue during mouse usage (Table 1). While flexion showed a statistically significant difference between keyboard and mouse tasks, the difference was less than 5°, suggesting that its contribution to symptom severity may be minimal compared to the effects of prolonged static posture. The smaller total distance moved during mouse tasks indicates that users tend to fix their head positions more while using a mouse than during keyboard tasks because users should focus their gaze on the cursor when using a mouse. This prolonged static posture likely imposes greater mechanical stress on the cervical spine, resulting in more severe clinical symptoms. The current study’s results support the theory that maintaining a fixed neck posture for a prolonged period can be a non-physiological state [31], which may increase mechanical stress on the spine. This is consistent with the results of a recent study showing that static postures, especially sitting during smartphone usage, were associated with higher discomfort scores than dynamic postures [32]. Mousavi-Khatir et al. [33] also reported that 10 min of prolonged static neck flexion decreased mechanical and neuromuscular responses of the cervical spine, potentially leading to spinal instability and injury. The 20 min of prolonged static posture in our study may have been sufficient to desensitize mechanoreceptive afferent neurons in soft tissue and intervertebral discs, resulting in altered cervical reflexive responses. Supporting this, another study showed that sustained static posture affected the median frequency on electromyography, considered a muscle fatigue index [34], further linking sustained static posture with muscle fatigue and the development of clinical symptoms. Considering the total distance moved during the task as a potential parameter of evaluating static posture, maintaining physiological movement through postural changes during work could be necessary to reduce neck symptoms.

In this study, all clinical symptoms were significantly more severe during mobile device use compared to desktop computer use and were associated with flexion and anterior translation. Interestingly, no distinct symptom patterns were observed based on task type or kinematic parameters. Given that the participants were young adults without a history of neck disorders, it is plausible that clinical symptoms were more reflective of muscle fatigue, manifesting as discomfort or fatigue, rather than mechanical irritation, such as sharp pain. Previous research on the qualitative description of neck pain in adults with a mean age of 33 years reported that 60% experienced stiffness and 13% reported sensations of heaviness or squeezing [27]. This aligns with our finding that discomfort and fatigue were significantly higher during the more static mouse task compared to the keyboard task. Neck symptoms can vary depending on the underlying causes; for example, sensory symptoms such as tingling in a nerve root distribution are common in patients with radiculopathy [35]. Thus, a different population might exhibit distinct symptom patterns depending on the specific underlying pathology.

Considering the results of this study, mitigating the risk of neck-related symptoms associated with prolonged device use requires ergonomic interventions that minimize excessive neck flexion, anterior translation, and static postures. Recommendations include prioritizing desktop computer use over mobile devices when possible, using smartphone holders to elevate devices to at or above the eye level, and incorporating regular posture changes and short breaks during tasks to promote dynamic movement and reduce muscle fatigue [36,37].

Despite these insights, there are several limitations to this study that warrant discussion. First, the total number of participants was small, and they were predominantly young and male, which may limit the generalizability of the findings to older adults, women, or individuals with a history of neck disorders, as the prevalence and nature of neck symptoms may differ across these populations [38,39]. Second, this study identified significant associations between individual kinematic variables and clinical symptoms but did not establish a predictive model incorporating multiple kinematic and kinetic variables or accounting for temporal variations in posture. Because continuous kinematic data were reduced to mean values over the recording period, this approach may not fully capture dynamic posture changes. Applying more advanced statistical methods, such as inferential analysis based on confidence bands [40], to assess continuous movement patterns over time could improve our understanding of their impact on clinical symptoms and provide insights for ergonomic interventions. Third, anthropometric variables, including weight, and BMI, as well as habitual device usage patterns, such as daily computer and mobile device use, which could influence cervical spine kinematics and clinical symptoms, were not assessed. Fourth, since the experimental environment was designed to replicate an adaptable workstation in non-ergonomic settings, certain ergonomic criteria, such as chair height adjustments, were not strictly controlled. Additionally, no restrictions were placed on participants’ head movements or gaze direction during the tasks, meaning that natural variations in posture may have influenced the results. Furthermore, while this study separately analyzed the effects of keyboard and mouse use on cervical posture and clinical symptoms, most people use both devices together and for longer durations in real-world scenarios, which may limit the generalizability of the findings. However, the decision to analyze these tasks separately was made to isolate and evaluate the specific effects of each device on neck posture and symptoms, providing a clearer understanding of their individual contributions. This approach allows for more targeted ergonomic recommendations, though future studies should aim to assess the combined use of both devices to reflect more typical working conditions.

## 5. Conclusions

The use of mobile devices was associated with greater neck flexion and anterior translation compared to desktop computer tasks, as well as significantly higher levels of all clinical neck symptoms. Moreover, these symptoms demonstrated a significant positive correlation with both flexion and anterior translation. Although further studies including kinetic evaluations and a wider range of conditions are warranted, this study highlights the importance of avoiding prolonged flexed neck positions, forward head posture, and static postures to minimize mechanical stress on the cervical spine and potentially reduce the risk of neck-related symptoms.

## Figures and Tables

**Table 1:** Clinical neck symptoms after tasks using mobile devices (smartphone and tablet) and desktop computer (keyboard and mouse).

	Mobile Device				Desktop Computer		
	Smartphone	Tablet	Total	Keyboard	Mouse	Total	*p*-Value
Discomfort	40.0 (29.8,50.1)	36.5 (26.3,46.7)	39.1 (30.4–47.8)	30.7 (20.3,41.0) ^a^	37.0 (26.6,47.3) ^a^	33.0 (24.3–41.7)	0.036 *
Pain	37.4 (25.5,49.3)	36.3 (24.4,48.2)	37.5 (29.3–45.6)	24.7 (15.2,34.2)	27.2 (17.7,36.7)	25.3 (17.2–33.5)	0.002 *
Tension	49.6 (37.4,61.7)	45.0 (32.8,57.2)	47.6 (38.3–57.0)	35.3 (25.6,45.0)	38.2 (28.4,47.8)	36.3 (27.0–45.6)	<0.001 *
Fatigue	47.8 (37.2,58.4)	51.4 (40.8,62.0)	49.6 (40.4–58.8)	33.9 (22.1,45.6) ^a^	42.8 (31.1,54.5) ^a^	38.3 (29.2–47.5)	0.005 *

All data are presented as least squares means (95% confidence intervals). Total values represent the adjusted averages derived from the linear mixed model, accounting for pre-task covariates (for discomfort, pain, and tension) and post-task values (for fatigue). * *p*-values < 0.05 indicate significant differences between mobile device and desktop computer usage via a linear mixed model. ^a^ Statistically significant difference between keyboard and mouse within the desktop computer task (*p*-value < 0.05).

**Table 2:** Mechanical neck symptoms in association with neck postures during mobile device or desktop usage tasks.

	Discomfort			Pain			Tension			Fatigue		
	ß	SE	*p*-Value	ß	SE	*p*-Value	ß	SE	*p*-Value	ß	SE	*p*-Value
Flexion	0.146	0.065	0.031 *	0.224	0.092	0.019 *	0.209	0.077	0.010 *	0.261	0.089	0.006 *
Lateral bending	−0.065	0.575	0.910	−0.063	0.773	0.936	0.325	0.688	0.639	0.037	0.782	0.962
Axial rotation	0.640	0.460	0.171	0.675	0.642	0.299	0.120	0.567	0.833	0.715	0.645	0.274
Anterior translation	0.263	0.088	0.005 *	0.358	0.122	0.005 *	0.281	0.105	0.011 *	0.289	0.124	0.024 *
Distance moved	−2.361	15.353	0.879	31.364	20.431	0.132	18.900	0.139	0.305	−2.202	21.060	0.917

SE: Standard Error. * *p*-value < 0.05 indicates significant association of a neck posture with a clinical neck symptom, independent of type of tasks, by linear mixed model analysis.

## Data Availability

The data that support the findings of this study are available from the corresponding author upon reasonable request.
